# Focus on Multidisciplinary Aspects of Sleep Medicine

**DOI:** 10.3390/brainsci13091327

**Published:** 2023-09-15

**Authors:** Alessandro Cicolin, Luigi Ferini-Strambi

**Affiliations:** 1 Regional Sleep Medicine Center, Department of Neurosciences Rita Levi Montalcini, University of Turin, 10126 Torino, Italy; 2 AOU Città della Salute e della Scienza, Department of Neurosciences and Mental Health, 10126 Torino, Italy; 3 Faculty of Psychology, Vita-Salute San Raffaele University, 20132 Milan, Italy; ferinistrambi.luigi@hsr.it; 4 Sleep Disorders Center, Division of Neuroscience, IRCCS San Raffaele Hospital, 20132 Milan, Italy

Sleep is an essential biological requirement for human life, alongside food, water, and air. Therefore, it is not surprising that insufficient sleep, in terms of both quantity and quality, may disrupt cognitive and physical performance, quality of life and health. Sleep is compromised in many cardiological, neurological, psychiatric, metabolic, pneumological, and oncological diseases, but it is also well known that sleep disorders significantly increase the risk of cardiovascular, neurological, and oncologic disease, and that they can deeply modify the outcome of coexisting medical conditions [1,2,3,4]. The consequences of undiagnosed and untreated sleep disorders result in a significant economic burden, represented by associated healthcare costs and indirect financial costs, mainly due to absenteeism, reduced productivity, as well as work-related and traffic accident [5,6].

In the light of the reciprocal relationship between sleep and health, it appears crucial that pulmonologists, endocrinologists, neurologists, cardiologists, otolaryngologists, psychiatrists, psychologists, pediatricians, dentists, and family medicine practitioners evaluate sleep quantity and quality in their daily activities.

Following this perspective, the goal of this Special Issue of *Brain Sciences*, entitled “Multidisciplinary Aspects of Sleep Medicine”, was to provide additional insight into this topic, including relevant studies from experts in the field.

Sleep bruxism is a common behavior, with a prevalence estimated in 13% of the general population, characterized by pain in the masticatory muscles and/or tooth wear. In a preliminary study, Wieckiewicz et al. [7] found a significant effect of opipramol (an atypical tricyclic antidepressant) in reducing bruxism episodes in subjects affected by severe bruxism, probably by reason of its effect on sigma receptor (agonism), dopamine receptors (antagonism), and histamine H1 receptors (antagonism).

Obstructive sleep apnea (OSA) is a multifactorial disease that is an increasing social and health problem occurring in about 3–10% of general population. It is characterized by recurrent breathing pauses due to the collapse of the upper airways and represents an independent risk factor for hypertension, ischemic heart disease, strokes, heart failure, atrial fibrillation, sudden cardiac death, cancer, and cognitive disorders. Urbanik et al. [8] analyzed the relation between the occurrence and the severity of OSA, and the ECG Holter monitoring findings in a group of patients with diagnosed OSA; they found that the severity of OSA (in terms of apnea–hypopnea index (AHI)) is an independent risk factor that predicts an increased number of supraventricular arrhythmias with a significant impact on the occurrence of conduction defects.

In the same area, Macek et al. [9] assessed the functional, structural changes of the right ventricular in patients with OSA using strain parameters and sleep structure. They observed a significant positive correlation between obstructive apnea severity and average free wall strain, snore and mid-free wall strain, average heart rate, basal free wall strain, and, finally, the frequency of Cheyne–Stokes breathing with basal septal strain and mid septal strain, highlighting the relationship between polysomnographic and echocardiographic findings.

In industrialized countries, professionally active populations frequently have reduced sleep that may lead to daytime sleepiness, cognitive deficits, and impaired response of the stress-related activities of the hypothalamic–pituitary axis and the autonomic nervous system. Gomez-Merino and colleagues [10], in their study, compared daytime sleepiness, cognitive performances, and salivary stress biomarker responses in healthy volunteers following 5 days of sleep restriction with or without association with relaxation techniques during daytime naps and the auditory stimulation of sleep slow oscillations during nighttime sleep. They found a beneficial effect of these techniques despite them being in different domains and likely acting through stimulating effects on the autonomic nervous system, as shown by the change in salivary α-amylase levels.

In the last decade, several studies evaluated the physiological and pharmacological properties of melatonin and its crucial role in regulating the sleep/wake rhythm. Biggio et al. [11], in their review, summarize the recent clinical and basic research data related to the multifactorial effects of endogenous and exogenous melatonin on different physiological (pregnancy, brain development, and aging), pharmacological (action through specific receptor complexes), and pathological (sleep disturbance and other diseases) conditions. The authors concluded that exogenous melatonin, in particular the prolonged release formulation, may be effective in reinstating not only the altered sleep/awake cycle, but also the reduced neuroprotective mechanisms during such uncomfortable conditions.

The content of this Special Issue may represent a preliminary, though still meaningful, contribution to the scientific evidence. Many research efforts in sleep medicine are moving towards novel approaches and new advancements for the diagnosis and treatment of sleep disorders. Relevant progress in the identification of the sleep functions and the reciprocal relationships between sleep disorders and systemic diseases may improve quality of life as well as the effectiveness of health care.
